# Comparative Analysis of Defects in Mg-Implanted and Mg-Doped GaN Layers on Freestanding GaN Substrates

**DOI:** 10.1186/s11671-018-2804-y

**Published:** 2018-12-11

**Authors:** Ashutosh Kumar, Kazutaka Mitsuishi, Toru Hara, Koji Kimoto, Yoshihiro Irokawa, Toshihide Nabatame, Shinya Takashima, Katsunori Ueno, Masaharu Edo, Yasuo Koide

**Affiliations:** 1 National Institute for Materials Science, Tsukuba, Ibaraki, 305-0047 Japan; 2 Fuji Electric Co., Ltd., Hino, Tokyo, 191-8502 Japan

**Keywords:** GaN, STEM, SIMS, Pyramidal defects, Line defects

## Abstract

Inefficient Mg-induced p-type doping has been remained a major obstacle in the development of GaN-based electronic devices for solid-state lighting and power applications. This study reports comparative structural analysis of defects in GaN layers on freestanding GaN substrates where Mg incorporation is carried out via two approaches: ion implantation and epitaxial doping. Scanning transmission electron microscopy revealed the existence of pyramidal and line defects only in Mg-implanted sample whereas Mg-doped sample did not show presence of these defects which suggests that nature of defects depends upon incorporation method. From secondary ion mass spectrometry, a direct correspondence is observed between Mg concentrations and location and type of these defects. Our investigations suggest that these pyramidal and line defects are Mg-rich species and their formation may lead to reduced free hole densities which is still a major concern for p-GaN-based material and devices. As freestanding GaN substrates offer a platform for realization of p-n junction-based vertical devices, comparative structural investigation of defects originated due to different Mg incorporation processes in GaN layers on such substrates is likely to give more insight towards understanding Mg self-compensation mechanisms and then optimizing Mg doping and/or implantation process for the advancement of GaN-based device technology.

## Introduction

Over the last three decades, GaN has emerged as one of the most investigated compound semiconductors all across the world. This is mainly due to its tremendous potential not only in solid-state lighting applications but also in high-power, high-frequency, and high-temperature operations [1–8]. For successful employment of devices based on GaN and related heterostructures into such applications and operations, controllable n-type and p-type doping is the key requirement. In this aspect, achieving and controlling n-type doping in GaN epilayers or single crystals are now optimized to a great extent in comparison to its p-type counterpart which is still a bottleneck for the academia as well as industry. Till now, Mg has proven to be the most efficient p-type dopant despite of its high activation energy which requires a large amount of Mg concentrations (around 10^19^ cm^−3^ or more) to be incorporated in order to achieve reasonable free hole concentrations close to 10^18^ cm^−3^. Any increase in Mg atomic concentrations beyond 10^19^ cm^−3^ lead to a decrease in the free hole concentration [9–11]. This phenomenon is mainly attributed to the creation of N vacancies [12–14], Mg-related point defects [10, 15], or Mg vacancies-related charged and/or neutral complexes which lowers the Fermi level and saturates free hole concentrations [16, 17]. On the basis of photoluminescence measurements giving rise to a peak at 2.9 eV, a deep donor defect complex Mg-V_N_ was also believed to be the one of the primary reason for the self-compensation mechanism. [17–19]. Hence, despite of the number of significant research attempts made towards understanding the Mg incorporation in GaN, this problem remains still unclear and further analysis needs to be carried out.

Most of the earlier reports on defects analysis using atomic scale microscopic studies are based on Mg-doped GaN layers grown on sapphire using metal organic chemical vapor deposition (MOCVD) or molecular beam epitaxy (MBE). To the best of our knowledge, there are only few reports on defects analysis in Mg-doped freestanding GaN substrates and there is no report on transmission electron microscopy-based defects analysis in freestanding GaN substrates where Mg is incorporated via ion-implantation. It is now well accepted that freestanding GaN substrates have several advantages over MOCVD/MBE grown GaN layers on foreign substrates due to reduced level of dislocation densities and their applications in efficient vertical devices. Achieving sufficient p-type dopants activity in such substrates via ion-implantation needs to be explored for commercialization and development of GaN-based solid-state lighting and high-power devices. Keeping all these issues as primary objective, we have carried out detailed structural analysis of Mg-incorporated defects in GaN freestanding layers where Mg incorporation is achieved by doping as well as ion implantation.

## Experimental Methods

Freestanding n-GaN substrates grown using hydride vapor phase epitaxy are used in our study. Thereafter, epitaxial layers are grown by MOCVD on these substrates. Mg incorporation is carried out via two approaches: ion implantation and epitaxial doping. In the first case, Mg is implanted into 4-μm-thick undoped GaN epitaxial layer without depositing any protection layer on undoped GaN layers, while in the second case, 1-μm-thick Mg-doped GaN is epitaxially grown on 4-μm-thick undoped GaN layers continuously. For a reliable comparison, level of Mg incorporation is kept same as 4 × 10^19^ cm^−3^ in both cases. The Mg implantation is carried out at 500 °C followed by annealing at 1350 °C for dopants’ activation. The implantation energies are taken as 15, 30, 55, 95, and 180 keV with respective dosages of 3.0 × 10^13^, 5.5 × 10^15^, 1.1 × 10^14^, 1.9 × 10^14^, and 8 × 10^14^ cm^−2^, to obtain a 200-nm-deep box profile. Hall measurements are carried out to evaluate the electrical properties of both Mg-doped and Mg-implanted GaN samples. For Mg-doped GaN sample, hole concentration and mobility are found to be 3.4 × 10^17^ cm^−3^ and 9.5 cm^2^/V-s. On the other hand, electrical properties of Mg-implanted sample could not be evaluated properly due to its highly resistive nature. The distribution of Mg as a function of depth is investigated using secondary ion mass spectrometry (SIMS) while scanning transmission electron microscopy (STEM) analysis is used for structural investigations of Mg-induced defects. For this, STEM and electron energy dispersive X-ray spectroscopy (EDS) have been carried out by JEOL JEM-ARM200F operated at 200 and 80 kV. For these studies, TEM specimens were prepared by focused ion-beam milling using Ga beams followed by a liquid nitrogen-cooled low-energy Ar ion milling.

## Results and Discussion

### Defects Analysis in Mg-Implanted GaN Layer

**Fig. 1:**
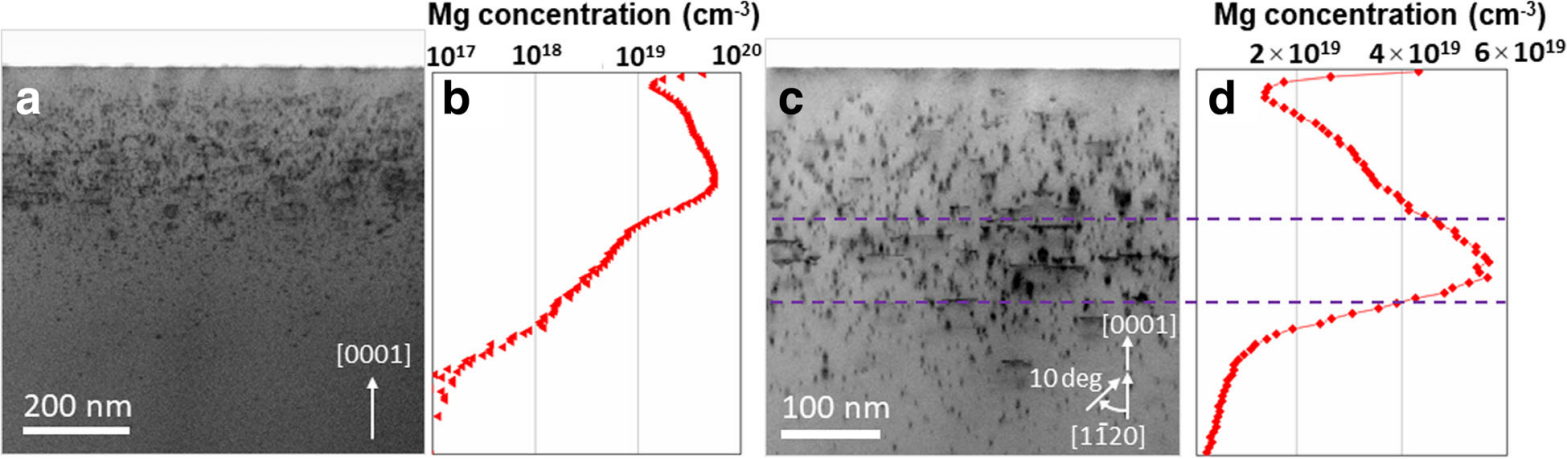
**a** Cross sectional bright field STEM image of Mg implanted GaN sample obtained along [11$\overline{2}$0] axis and **b** corresponding depth profile of Mg obtained using SIMS. For better visualization of the defects and their relation to Mg concentration, imaging is carried out under off-zone axis condition as shown in **c**. Mg profile corresponding to **c** is shown in **d** in linear scale where line defects are observed in a narrow region having highest Mg concentration

Figure 1(a) shows the bright field (BF)-STEM image of Mg-implanted GaN whereas (b) shows the corresponding SIMS profile. The arrow shown in Fig. 1(a) represents positive [0001] direction, and images are viewed along [11$\overline{2}$0] zone axis. It can be seen that defects are not uniformly distributed as a function of depth, in fact, a direct correlation is seen between the concentrations of Mg and defects. Most of the defects are accumulated at about 150 nm from the surface where Mg concentration is more than 10^19^ cm^−3^ as observed from SIMS measurements. For better understanding of defects and their visualizations due to Mg implantation in GaN, imaging is carried out in off-zone axis condition by tilting the sample 10° around c-axis from [11$\overline{2}$0] axis. This condition weakens the diffraction contrast due to perfect crystal and enhances the defects contrast which allows better visualization of the defects in comparison to surrounding environment. BF-STEM image of Mg-implanted GaN sample taken under this off-zone axis conditions is shown in Fig. 1(c) where some line defects are seen at a depth of about 200 nm from the surface. The corresponding Mg SIMS profile is presented in (d) in linear scale where a direct correspondence is observed between the existence of these line defects and Mg concentration. These defects are found to be located in a narrow region where Mg concentration is about mid 10^19^ cm^−3^ range.

**Fig. 2:**
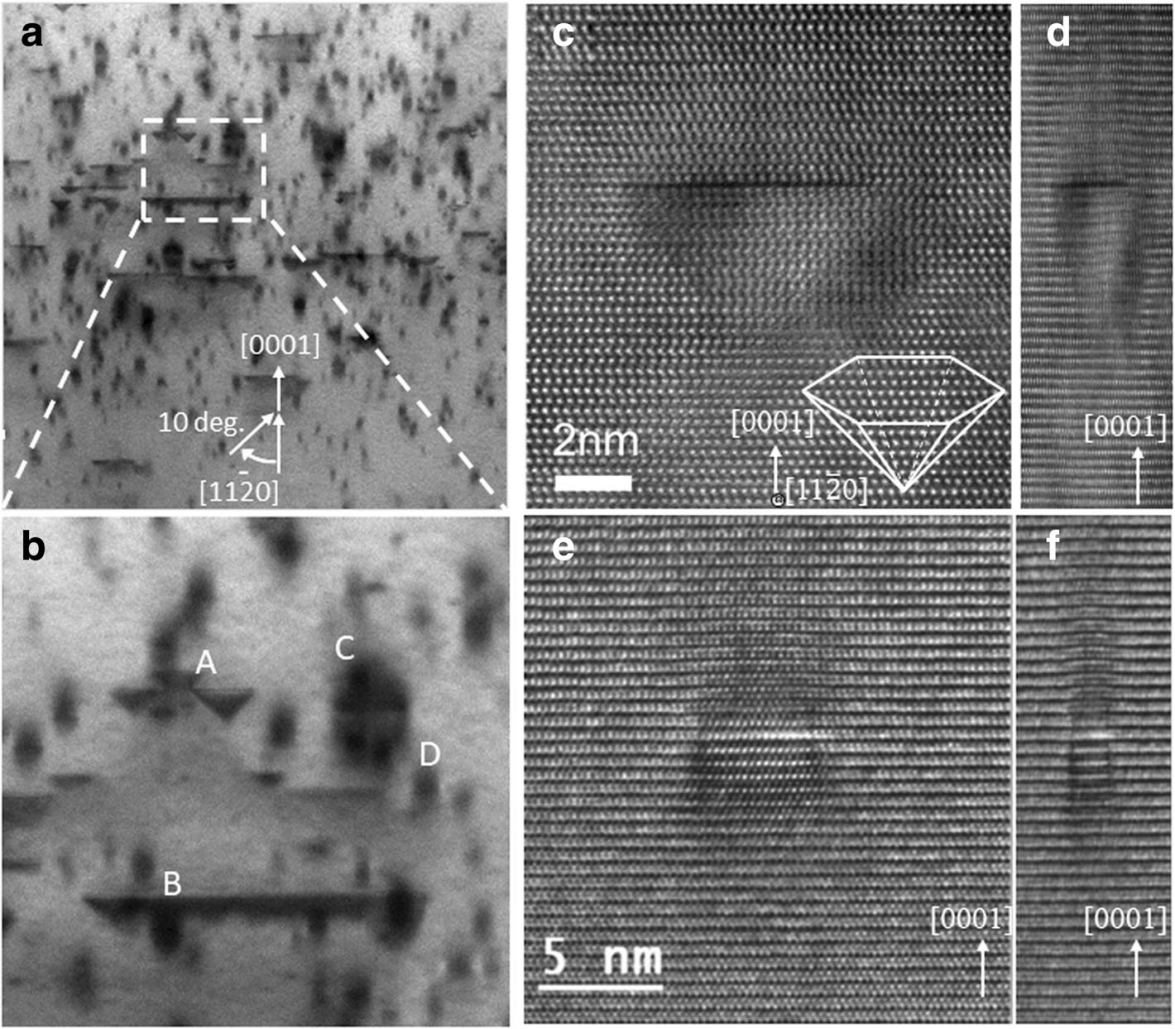
**a** Cross sectional bright field STEM image of Mg implanted GaN sample obtained in off-zone axis condition to strengthen the defects contrasts. Magnified view of marked region in (**a**) is presented in (**b**) where four different kind of defects labelled as A, B, C and D are observed. High-resolution TEM image of the pyramidal domains labelled as A in (**b**) is shown in (**c**) with schematic representation at right bottom. The distorted lattice on the pyramidal domain are representing by squeezing (**c**) as shown in (**d**). High-resolution TEM image of one typical C or D type defects is presented in (**e**) with squeezed image in (**f**), showing strain field contrast around the defect

Further, high-magnification BF-STEM imaging is carried out under off-zone axis condition as shown in Fig. 2(a) with (b) showing the selected region of (a) at a higher magnification. As shown in (b), four kinds of structures labelled as A, B, C, and D are observed. The defects labelled as “A” are of pyramidal shape whereas “B” appear as line defects.

The structural observations of these defects are important from view point of understanding Mg compensation mechanisms, and next sections of the manuscript are mainly devoted to structural analysis of type “A” and “B” defects. The structures shown as “A” are pyramidal defects with positive [0001] direction pointing towards their head, their base on [0001] plane with six walls on [11$\overline{2}$3] planes inclined as shown by high-resolution TEM image of one such defect in Fig. 2(c). The schematic representation of such pyramidal domain is also shown in Fig. 2(c). Figure 2 (c) is squeezed perpendicular to [0001] direction as shown in (d) where lattice appears to be distorted in pyramidal domain in comparison to surrounding GaN matrix, indicating displacement between the Ga and N sublattices inside and outside of these pyramidal domains. This is consistent with the findings of the Vennegues et al. [20] where similar type of pyramidal domains are observed. The similar pyramidal shape defects are earlier observed in Mg-doped GaN films and their existence is usually explained by modification in GaN atomic structure due to Mg introduction [19–24]. Liliental-Weber et al. [25, 26] proposed that such pyramidal defects originate from Mg-rich clusters present near the head of these pyramids. The GaN structure in wurtzite phase is usually described by hexagonal stacking of N planes with half of the N sublattice tetrahedra sites filled by Ga atoms. Vennegues et al. [27] on the basis of their investigations proposed that introducing higher level of Mg in GaN results in the substitution of Ga by Mg, forming Mg_3_N_2_, a Mg-N compound reported to have antibixbyite structure. The antibixbyte structure of Mg_3_N_2_ corresponds to the filling of N sublattice tetrahedra sites by Mg which occupies three out of every four sites. As per the model proposed by Vennegues et al. [27], a pyramidal domain can be considered as two GaN crystals of opposite polarity separated by a monolayer of Mg_3_N_2_. This is further supported by the investigations of Hansen et al. [28] where these pyramidal domains were proposed to be Mg_3_N_2_ inclusions. Vennegues et al. [27] and Leroux et al. [23] also reported that formation of such pyramidal domains having nanometer size also requires a Mg incorporation of low to mid 10^19^ cm^−3^ range. This is consistent with our findings where the pyramidal shape defects are observed at lower to mid-10^19^ cm^−3^ Mg concentrations as seen from the correlation between STEM image (Fig. 1(c)) and corresponding SIMS profile (Fig. 1(d)). Therefore, the pyramidal shape defects labelled as structures “A” in Fig. 2 (b) of our study are believed to be Mg-rich pyramidal domains and their formation can be directly linked to the Mg compensation mechanism in p-GaN layers. Other types of defects as shown in Fig. 2(b) are type “C” and “D” defects which are essentially similar structures with variation in their dimensions. The contrast which appears to be elongated along the [0001] direction when viewed from [11$\overline{2}$0] axis, is likely to be strain originated. For further clarifying this, high-resolution TEM image of one such similar defect is presented in Fig. 2(e) with (f) showing the same image squeezed perpendicular to [0001] direction. The distorted lattice along [0001] direction suggests different lattice constant due to different strain field along this direction. As Mg is smaller in size in comparison to Ga, its incorporation on the Ga sites is expected to produce strain in the lattice which may lead to this contrast around these defects.

**Fig. 3:**
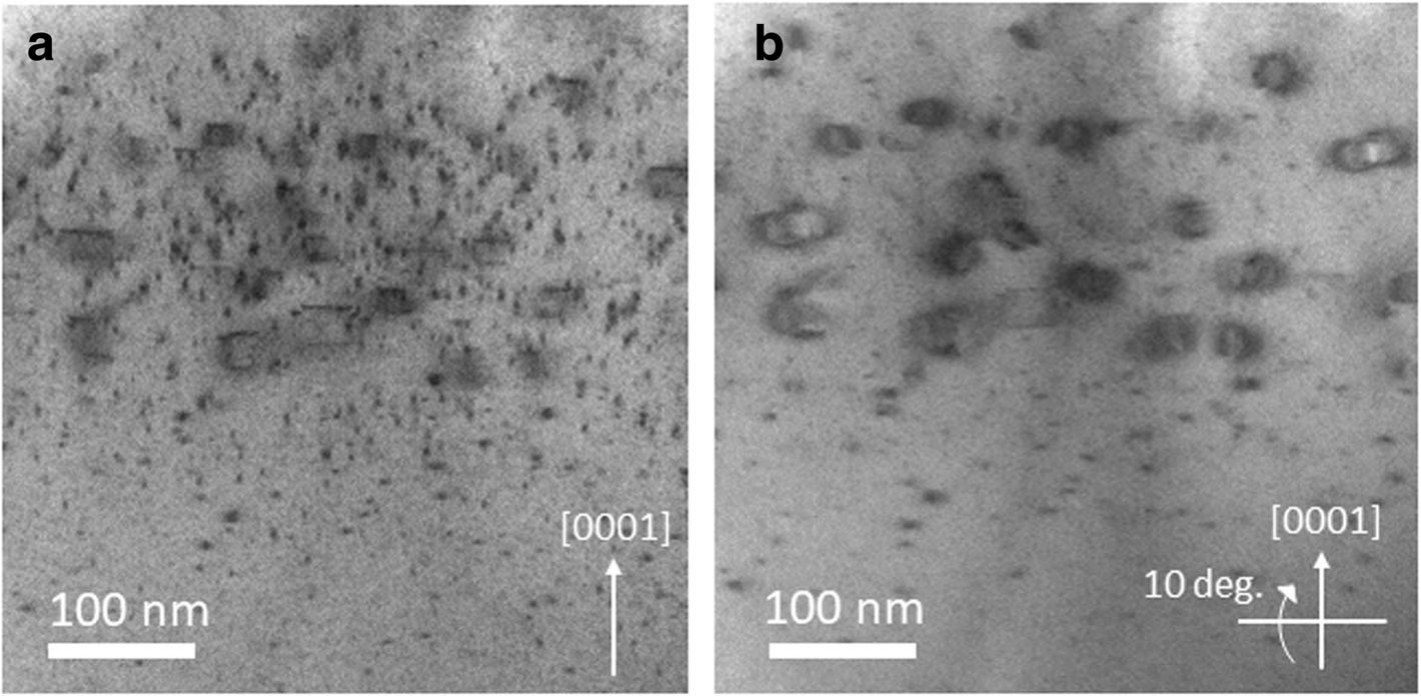
**a** Cross-sectional bright field STEM image of Mg-implanted bulk GaN sample for analyzing type B defects which appear like truncated pyramids or trapezoidal shape. **b** represents the image taken by tilting the sample at 10° around the axis perpendicular to *c*-axis where a different contrast is observed at the edges of these defects

Another type of defects, labeled as “B” in Fig. 2 (b) appear to be line defects perpendicular to [0001] direction, when viewed from [11$\overline{2}$0] axis. It is important to note that such type of defects appear to be accumulated in a narrow region having higher Mg concentrations (as observed from correspondence between BF-STEM image and Mg SIMS profile shown in Fig. 1(c) and (d)) which suggests that their formation is associated with the over-incorporation of Mg. Another observation is the presence of the pyramidal domains on the edges of these line defects which indicate that accumulation of these domains may result in their formation. However, one should not rule out that it could simply be a random overlap of pyramidal and line defects and further investigations are needed in this direction. The BF-STEM image showing these types of defects is presented in Fig. 3(a). For better understanding of these defects, sample is tilted at about 10° around the axis perpendicular to [0001] direction and obtained BF-STEM image is shown in Fig. 3(b). This tilting of the sample strongly excites the diffraction spots along 1–100 direction, which results in enhanced contrast from the strain field in the direction surrounding the defect. From this strain field contrast, the defect which appeared as the line (see Fig. 2(a) and (b)) actually consists of a pair of lines separated by few nm, deeper in [0001] direction.

**Fig. 4:**
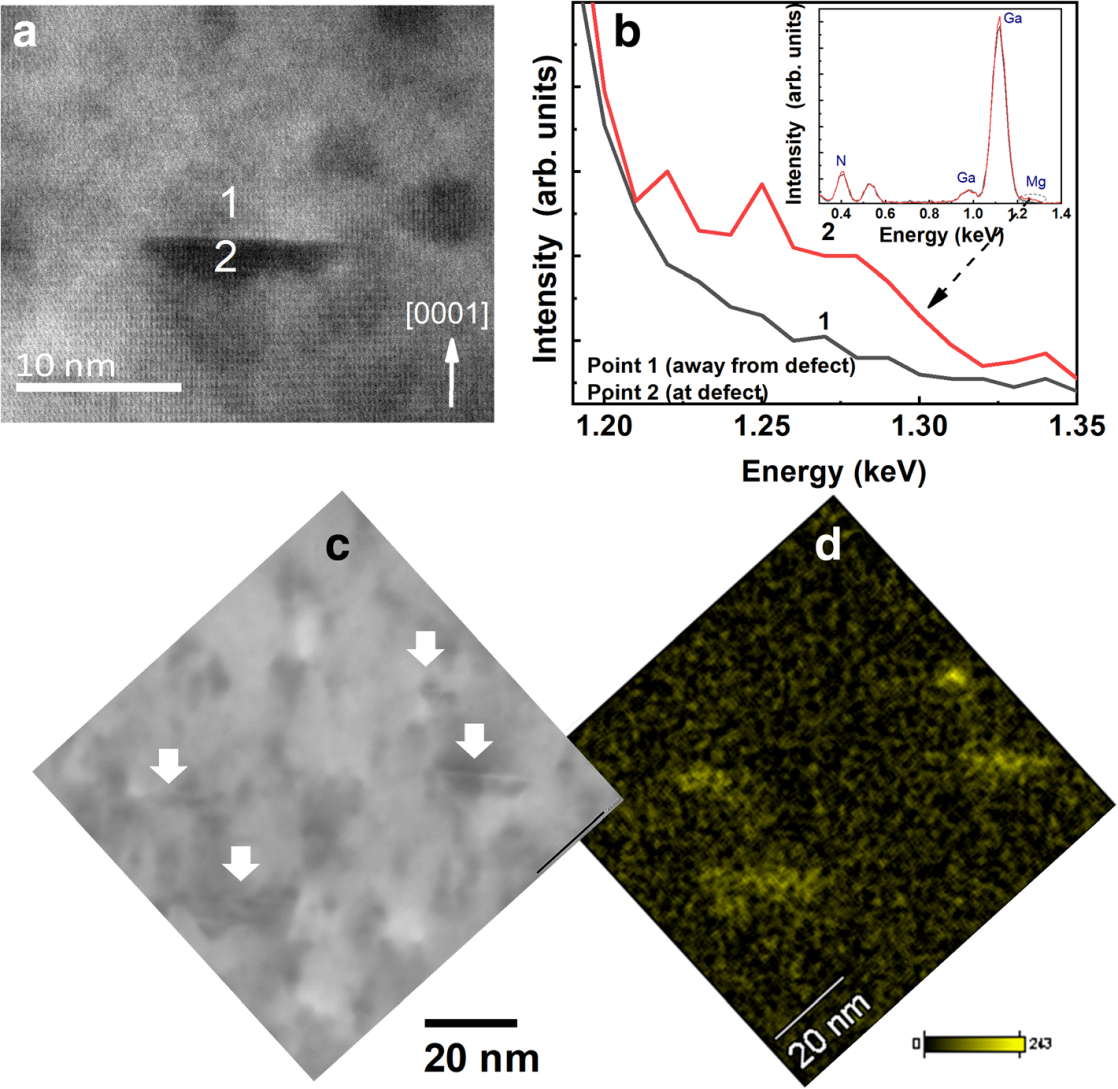
**a** Cross-sectional STEM image of Mg implanted GaN sample showing an individual type “B” defect. Points 1 and 2 represents regions where EDS measurements are carried out and resulting EDS spectra are shown in **b**. EDS spectra in the energy range of 1.19–1.40 keV is plotted in **b** with inset showing the full EDS spectra with Ga and N peaks. **c** and **d** present STEM image and corresponding Mg map of a similar Mg-implanted bulk sample showing the presence of Mg in these types of defects

There could be a possibility of presence of Mg in these defects as they appear in the narrower region where Mg concentration is higher than 10^19^ cm^−3^ as observed from the correspondence between STEM image (Fig. 1(c)) and Mg SIMS profile (Fig. 1(d)). To validate this belief of these types of defects having Mg, we carried out scanning transmission electron microscopy-energy-dispersive spectroscopy (STEM-EDS) measurements with EDS probe diameter less than 0.2 nm, at two different regions: “away from defect” and “at defect” labelled as points 1 and 2 respectively as shown in Fig. 4(a). The comparative EDS spectra from points 1 and 2 in the energy range of 1.19 keV to 1.35 keV where Mg peak is expected are plotted in Fig. 4(b) with inset showing full EDS spectra. The presence of Mg is clearly seen at defect (point 2). To further justify this, we have carried out STEM-EDS mapping on a similar Mg-implanted GaN sample. Figure 4 (c) presents STEM image of Mg-implanted GaN sample with downward arrows showing these line defects, and the corresponding EDS map of Mg is shown in Fig. 4 (d). The presence of Mg is clearly seen in these defects. Therefore, these defects contain Mg and their formation at Mg concentrations higher than 10^19^ cm^−3^ is likely to be another cause of Mg compensation.

### Defects Analysis in Mg-Doped GaN Layer

**Fig. 5:**
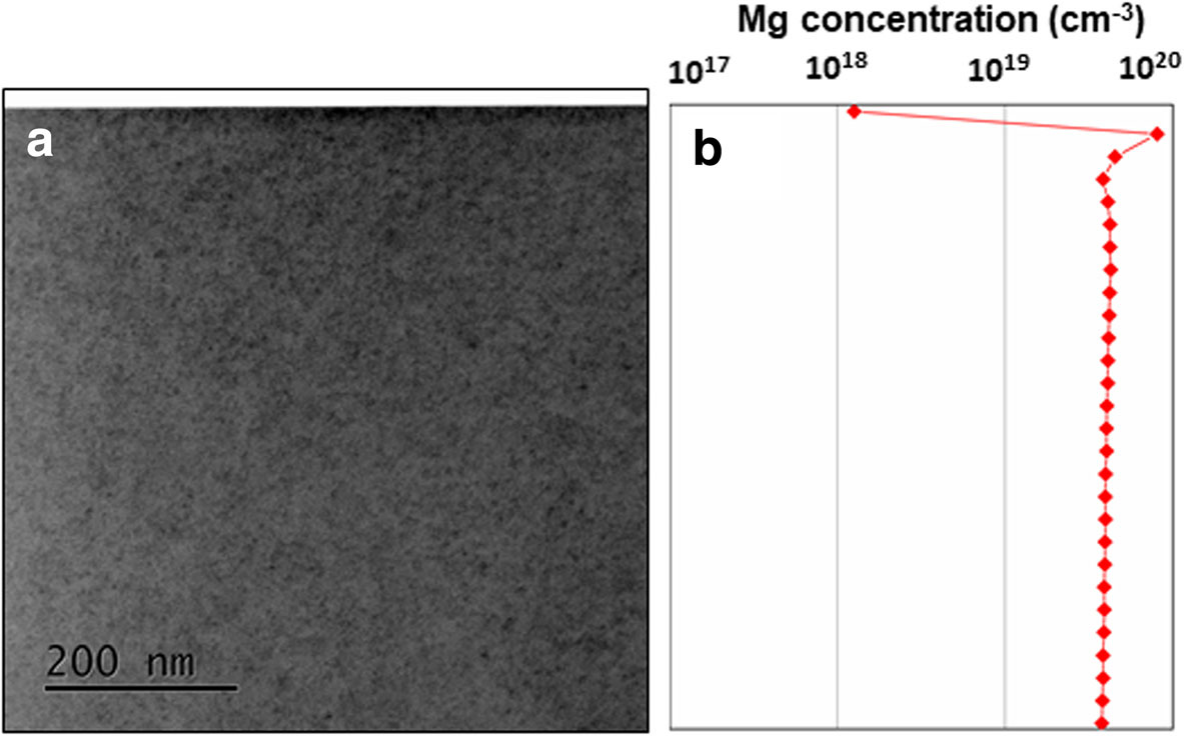
**a** Cross sectional bright field STEM image of Mg-doped bulk GaN sample obtained along [112 ®0] axis and **b** corresponding depth profile of Mg obtained using SIMS. Dot-like defects are found to be uniformly distributed across the sample

Next, we have carried out structural investigations on the GaN sample where 1-μm thick Mg-doped GaN layer is epitaxially grown on 4-μm-thick undoped GaN epitaxial layer. It is worth mentioning again that the level of Mg is kept same, i.e., 4 × 10^19^ cm^−3^ for a meaningful comparison between two approaches of Mg incorporation: epitaxially doped and ion implantation. Figure 5(a) shows BF-STEM image of Mg-doped GaN grown epitaxially on freestanding GaN substrates, viewed along [11$\overline{2}$0] whereas (b) shows Mg profile as a function of GaN depth obtained using SIMS. Note that Mg concentration remains almost constant at about 4 × 10^19^ cm^−3^ within the field of view of Fig. 5(a) (up to 700 nm) unlike the earlier case of Mg-implanted GaN where Mg concentration was found to be a function of GaN depth (see Figs. 1 (a)–(d)).

**Fig. 6:**
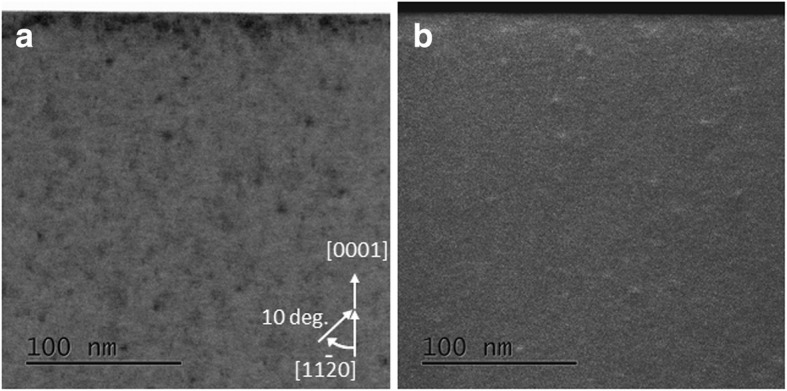
Cross-sectional **a** STEM-BF and **b** STEM-ADF images of Mg-doped bulk GaN sample obtained in off-zone axis condition to strengthen the defects contrasts

To carry out defects analysis in Mg-doped GaN sample, STEM imaging in off-zone axis condition by tilting the sample at 10° around *c*-axis from [11$\overline{2}$0] axis is carried out. Figures 6 (a) and (b) represents the BF-STEM and DF-STEM images where dot like defects having size of about 5 nm are observed to be uniformly distributed across the sample. Note here that Mg profile also appears to be uniform in this case as observed from SIMS profile (see Fig. 5(b)). The uniform distribution of Mg and these defects across the GaN sample suggests a direct correlation between these defects and Mg incorporation. These dot like defects of about 5 nm are likely to be precipitates of Mg (and possibly induced stacking faults by it). Due to their small size, Mg precipitation itself could not be directly confirmed by EDS measurements (Ga and Mg peaks lie quite close to each other which makes mapping of small concentration difference extremely difficult).

This observation is completely different from the earlier case of Mg-implanted sample where defects are found to be accumulated at 200 nm from the surface where Mg concentration was maximum. In addition, unlike the Mg-implanted sample, we did not observe any pyramidal and two-line defects, (labelled as A and B in Fig. 2(b)) in Mg-doped GaN sample. Interestingly pyramidal and line defects are also reported in the GaN samples where Mg is incorporated by techniques other than ion-implantation. For example, Khromov et al. [29] reported the existence of pyramidal defects in Mg-doped GaN samples grown by MOCVD. However, they observed such defects only in higher doped GaN sample where Mg concentration was about ~ 5 × 10^19^ cm^−3^. However, in samples with Mg ~ 2 × 10^18^ cm^−3^, these pyramidal domains were not observed. Vennegues et al. [27] also observed such pyramidal domains in MOCVD grown Mg-doped GaN samples with Mg concentrations lying in the mid 10^19^ cm^−3^ range. They did not observe such defects in sample with Mg concentrations lower than 10^19^ cm^−3^. In our work, similar level of Mg ~ 4 × 10^19^ cm^−3^ is incorporated via ion-implantation and epitaxial doping to analyze the presence of these defects. These defects are only observed in Mg-implanted sample, not in Mg-doped sample which suggests that Mg distribution should also be considered for explaining their existence. From SIMS measurements, Mg is found to be non-uniformly distributed in the Mg-implanted sample (Figs. 1(b) and (d)) whereas Mg-doped sample showed uniform distribution of Mg (Fig. 5 (b)). Moreover, in Mg-implanted sample, these defects were found to be existing only in a narrow window with higher Mg concentration in comparison to surrounding matrix. Therefore, formation of these defects is linked with the level of incorporated Mg and distribution of Mg and they are likely to be form in the regions where Mg lies in the range of 10^19^ cm^−3^. It appears that non-uniform Mg profile in Mg-implanted sample leads to a non-uniform distribution of defects. However, one should not deny the possibilities like non-uniformly distributed defects causing non-uniform Mg distribution or dependency of Mg implantation profile on existence of such defects; therefore, further investigations are needed in this direction. Our comparative analysis of defects in Mg-incorporated GaN suggests that the nature and type of the defects is dependent on the incorporation method.

## Conclusions

To summarize, scanning transmission electron microscopy-based structural investigations of defects in Mg-implanted and Mg-doped epi-layers on freestanding GaN substrates revealed that nature of defects depends strongly on the method of Mg incorporation. Mg-implanted GaN showed the presence of pyramidal domains pointing towards [0001] direction and two-line defects with features separated by few nanometers deeper in [0001] direction. Pyramidal domains are believed to be Mg_3_N_2_-based structures whereas line defects are also found to have Mg as observed from energy-dispersive spectroscopy. These line defects are found to be located at a depth of about 200 nm from the surface, in a narrow region having Mg concentration of about mid 10^19^ cm^−3^ which suggest that their formation is linked to the level of implanted Mg concentration. The formation of these defects in GaN on Mg implantation is expected to contribute significantly to Mg self-compensation mechanisms leading to inefficient p-type doping. On the contrary, Mg-doped GaN sample only showed the presence of dot-like defects which are found to be uniformly distributed across the whole sample. Present study highlighting the dependence of Mg incorporation method and its concentrations on the nature and type of defects may prove useful for choosing appropriate amount of Mg to be incorporated for achieving high p-type conductivity in GaN-based materials for efficient device operation.

## Abbreviations

ADF: Annular dark field
BF: Bright field
EDS: Energy-dispersive spectroscopy
MBE: Molecular beam epitaxy
MOCVD: Metal-organic chemical vapor deposition
SIMS: Secondary ion mass spectrometry
STEM: Scanning transmission electron microscopy
